# Cardiac Autonomic Functions May be Influenced by Body Weight

**DOI:** 10.5935/abc.20170154

**Published:** 2017-12

**Authors:** Mustafa Gulgun

**Affiliations:** Gulhane Training and Research Hospital - Department of Pediatric Cardiology, Ankara - Turkey

**Keywords:** Heart Rate, Diabetes Mellitus, Type 1, Young Adult, Risk Factors, Adolescents

**To the Editor,**

I read the paper by Silva et al.^1^ entitled ‘‘Sensitivity, Specificity and Predictive Value of Heart Rate Variability Indices in Type 1 Diabetes Mellitus’’ published in Arquivos Brasileiros de Cardiologia in March 2017. They aimed to compare Heart Rate Variability (HRV) indices and evaluate their sensitivity, specificity, and predictive value in young type 1 diabetic patients and healthy volunteers. It was showed a decrease in sympathetic and parasympathetic activities, as well as overall variability of autonomic nervous system in the diabetic group. I would like to congratulate Silva et al.^1^ for their valuable effort in this study. I also have a few comments to do.

The indices of HRV give us valuable numeric data about cardiac autonomic nerve system by processing RR interval variability beat-to-beat. A decrease in HRV implying an impaired autonomic cardiac function is one of the independent risk factors related to sudden cardiac death^1-4^. Several variables such as age, sex, obesity, drugs, ischemic heart disease, hypertension, hyperlipidemia, insulin resistance, might have effect on HRV indices^1-4^.

Body mass index is one of the most important influential factors on HRV indices^3^. Recently, a research reported decreased HRV because of inflammatory processes in the obese population^4^. In the study by Silva et al.,^1^ it is clearly seen that there is a statistically significant difference between the healthy group and the diabetic group in terms of body mass index. In addition, the blood lipid levels of the study population are not available in the study by Silva et al.^1^. In my opinion, the results of the study by Silva et al.^1^ might be more powerful if influential factors such as body mass index, blood lipid levels would have been considered. It would be more acceptable to make a comparison between the groups in which there was no statistically significant difference in terms of various influential factors such as body mass index. Thus, we could understand clearly the exact role of type 1 diabetes mellitus on the heart rate variability, which has prognostic importance in the sudden cardiac death.
