# Supplementary material for: Cardiac Autonomic Functions May be Influenced by Body Weight
Source: Arq Bras Cardiol. 2017 Dec;109(6):620–1. doi: 10.5935/abc.20170154 (PMC5783446; doi:10.5935/abc.20170154)
Supplement: Related texts

# Reply

**Authors:** Anne Kastelianne França da Silva, Diego Giuliano Destro Christofaro, Aline Fernanda Barbosa Bernardo, Franciele Marques Vanderlei, Luiz Carlos Marques Vanderlei

Initially we thank you for your interest in our article^1^ and the comments related to it. In fact, as pointed out, obesity exerts an important influence on autonomic cardiac modulation. Several studies published by our group, using heart rate variability (HRV) as a form of analysis, pointed out alterations in autonomic modulation in children^2-4^ and young obese.^5^ These studies demonstrate a reduction of parasympathetic, sympathetic and global activity, as well as a reduction in fractal correlation properties of heart rate^3^ in these individuals.

In this context, we understand the concern raised regarding the inclusion of individuals with high body mass index in the data set evaluated and we agree that this is an aspect that should be taken into account in the HRV analyzes. As can be seen in table 2 of the article,^1^ the data referring to HRV indices analyzes were adjusted for both body mass index and casual blood glucose. In this way, we can affirm that although there were significant differences for the body mass index and random blood glycemia between the groups, these did not influence the results obtained in our article.^1^

It is also important to note that of the 82 individuals evaluated, only six had a high body mass index (> 30 kg/m^2^) and although the body mass index had a statistically significant difference between the groups, the mean values were within the normality range and below those considered for both obesity and overweight.^6^

Regarding lipid measurements, unfortunately these data were not evaluated in our study, so it would not be appropriate to make any comments about it. We hope to have clarified the aspects raised and encourage further studies with this population addressing the limiting factors of our study.

**Sincerely,**

Anne Kastelianne França da Silva  Diego Giuliano Destro
Christofaro  Aline Fernanda Barbosa Bernardo  Franciele Marques
Vanderlei  Luiz Carlos Marques Vanderlei

## References

[1] Silva AK, Christofaro DG, Bernardo AF, Vanderlei FM, Vanderlei LC (2017). Sensitivity, specificity and predictive value of heart rate
variability indices in type 1 diabetes mellitus. Arq Bras Cardiol.

[2] Vanderlei LC, Pastre CM, Freitas Jr IF, Godoy MF (2010). Geometric indexes of heart rate variability in obese and
eutrophic children. Arq Bras Cardiol.

[3] Vanderlei LC, Pastre CM, Junior IF, de Godoy MF (2010). Fractal correlation of heart rate variability in obese
children. Auton Neurosci.

[4] Vanderlei LC, Pastre CM, Freitas Junior IF, Godoy MF (2010). Analysis of cardiac autonomic modulation in obese and
eutrophic children. Clinics (Sao Paulo).

[5] Rossi RC, Vanderlei LC, Gonçalves AC, Vanderlei FM, Bernardo AF, Yamada KM (2015). Impact of obesity on autonomic modulation, heart rate and
blood pressure in obese young people. Auton Neurosci.

[6] Associação Brasileira para o Estudo da Obesidade e
da Síndrome Metabólica (2016). Diretrizes Brasileiras de Obesidade.
